# Inhibitory Effects of a *Sargassum miyabei* Yendo on *Cutibacterium acnes*-Induced Skin Inflammation

**DOI:** 10.3390/nu12092620

**Published:** 2020-08-27

**Authors:** Mi-Jin Yim, Jeong Min Lee, Hyun-Soo Kim, Grace Choi, Young-Mog Kim, Dae-Sung Lee, Il-Whan Choi

**Affiliations:** 1 Department of Genetic Resources, National Marine Biodiversity Institute of Korea, Seocheon 33662, Korea; mjyim@mabik.re.kr (M.-J.Y.); lshjm@mabik.re.kr (J.M.L.); gustn@mabik.re.kr (H.-S.K.); gchoi@mabik.re.kr (G.C.); 2 Marine-Integrated Bionics Research Center, Pukyong National University, Busan 48513, Korea; ymkim@pknu.ac.kr; 3 Department of Microbiology and Immunology, College of Medicine Inje University, Busan 47392, Korea

**Keywords:** acne vulgaris, *Cutibacterium acnes*, *sargassum miyabei*, interleukin-8, activator protein-1

## Abstract

Acne vulgaris is a chronic inflammatory condition of skin sebaceous follicles. To explore its effects on acne vulgaris, we investigated the antibacterial and anti-inflammatory activities of *Sargassum miyabei* Yendo (a brown alga) ethanolic extract (SMYEE) on *Cutibacterium acnes* (*C. acnes*)-stimulated inflammatory responses, both in vivo and in vitro. To induce inflammation in vivo, *C. acnes* was intradermally injected into the dorsal skin of mice, to which SMYEE was applied. The antimicrobial activity of SMYEE was evaluated by the determination of minimum inhibitory concentrations (MICs). To explore in vitro anti-inflammatory effects, HaCaT cells were stimulated with *C. acnes* after treatment with SMYEE. The levels of IL-8 and the underlying molecular effects in *C. acnes*-stimulated HaCaT cells were assessed by enzyme-linked immunosorbent assay, Western blotting, and an electrophoretic mobility shift assay. Mouse skin lesions improved after treatment with SMYEE (50 μg/mouse). Neutrophil infiltration was significantly reduced in SMYEE-treated compared to SMYEE-untreated skin lesions. SMYEE reversed the *C. acnes*-induced increase in IL-8 levels in HaCaT cells and suppressed dHL-60 cell migration. SMYEE also inhibited *C. acnes*-induced phosphorylation of the extracellular signal-regulated kinase and inhibited activator protein-1 signaling. SMYEE may be a useful treatment for *C. acnes*-induced acne vulgaris.

## 1. Introduction

Acne vulgaris is a skin disorder involving sebaceous follicles. Its pathogenesis is poorly understood, but inflammation has been found to be a major contributor [1]. *Cutibacterium acnes* (*C. acnes*), a gram-positive anaerobic bacterium, triggers the inflammatory response of acne vulgaris by enhancing the secretion of interleukin-1 beta (IL-1β), IL-6, IL-8, and tumor necrosis factor-alpha (TNF-α) by monocytic cells, because of which inflammatory lesions eventually develop [2,3]. Keratinocytes are the first-line responders of the skin’s immune system and, along with sebocytes, produce a variety of cytokines and chemokines [4]. IL-8, a prototypical member of the CXC chemokine family, is a crucial inflammatory activator and is potently chemotactic for neutrophils [5]. IL-8 has been implicated in the inflammatory response of acne vulgaris [6]. *P. acnes* activates TLR-2 to induce the production of pro-inflammatory cytokines through activating MAP kinases (MAPKs), nuclear factor-κB (NF-κB), and activator protein (AP)-1 in facial acne lesions of patients [7].

*Sargassum*, a genus of brown seaweed, is widely distributed in the oceans and has been consumed as food and medicine in East Asia for centuries [8,9]. Numerous bioactive compounds, including meroterpenoids, carotenoids, phlorotannins, fucoidans, sterols, and glycolipids, have been identified from *Sargassum* [10]. Their pharmacological actions include anticancer, antibacterial, antifungal, antiviral, anti-inflammatory, antioxidant, and antimelanogenic effects [11]. Diverse pharmacological properties and structurally novel compounds may be the major contributors for the traditional therapeutical effects of *Sargassum* [9,12]. However, one species, *Sargassum miyabei* Yendo, has been studied only for its anti-inflammatory activities; any pharmacological effect on *C. acnes*-induced acne vulgaris remains unclear. Various antibiotics, chemical peels, and hormones have been used to kill the bacteria that trigger acne vulgaris-related inflammation [13,14,15,16]. However, these agents may lead to the emergence of resistant pathogens, and side-effects such as the emergence of drug resistant bacteria, immune hypersensitivity, and organ damage in the case of long-term usage [17,18]. Therefore, new agents with higher therapeutic efficacy and better safety profiles are required. Marine algae are a public health priority for exploring effective natural antimicrobial agents with better potential, fewer side effects than antibiotics, good bioavailability, and minimal toxicity [19]. Most studies on *C. acnes*-induced inflammatory cytokines were performed in murine models [3,20,21]. MIP-2 in mice is regarded as a functional homologue of IL-8 in humans, which, targeted against *C. acnes*, induces inflammation in mice [22]. Here, we examined the in vitro and in vivo effects of a *Sargassum miyabei* Yendo ethanolic extract (SMYEE) on *C. acnes*-induced inflammatory responses.

## 2. Materials and Methods

### 2.1. Reagents

Antibodies against lamin B (cat. no. sc-6217), goat anti-rabbit IgG–horseradish peroxidase (HRP) conjugate (cat. no. sc-2004), and NIMP-R14 (cat. no. sc-59338) were purchased from Santa Cruz Biotechnology, Inc. (Santa Cruz, CA, USA). Antibodies against ERK (cat. no. 9122), phospho(p)-ERK (cat. no. 2338), JNK (cat. no. 9252), p-JNK (cat. no. 9251), p38 MAPK (cat. no. 9212), p-p38 MAPK (cat. no. 9211), p-c-Jun (cat. no. 2361), and p-c-Fos (cat. no. 5348) were purchased from Cell Signaling Technology Inc. (Danvers, MA, USA). U0126 (cat. no. U120) was purchased from Sigma-Aldrich (St. Louis, MO, USA).

### 2.2. Preparation of a Sargassum Miyabei Yendo Ethanol Extract

The brown seaweed, *Sargassum miyabei* Yendo, was collected near Pohang, Korea, washed with tap water to remove slats, epiphytes, and sand, and stored at −20 °C. The frozen samples were lyophilized and homogenized using a grinder prior to extraction. The dried powder was extracted with 70% (*v*/*v*) EtOH (1:10 *w*/*v*) for 1 h (five repeats) with sonication, and the extract was evaporated to dryness in vacuo. The powder was dissolved in 70% (*v*/*v*) EtOH before use in bioactivity experiments.

### 2.3. Bacterial Strains and Culture Conditions

The type strain *C. acnes* KCTC 3314 was obtained from the Korean Collection for Type Cultures (KCTC; Daejeon, Korea) and *C. acnes* clinical isolates were provided by the Gyeongsang National University Hospital (Jinju, Korea), a member of the National Biobank of Korea. *C. acnes* strains were anaerobically cultured in the brain heart infusion broth (Difco, Detroit, MI, USA) with 1.0% (*w*/*v*) glucose in an anaerobic atmosphere created using the BBL GasPak system (Becton Dickinson Microbiology Systems, Cockeysville, MD, USA).

### 2.4. Minimum Inhibitory Concentrations (MIC) and Minimum Bactericidal Concentration (MBC)

The MICs were the lowest concentrations of the crude extract that inhibited visual growth after incubating aerobic bacteria for 18 h and anaerobic bacteria for 48 h [23]. The MICs of SMYEE were determined by using the two-fold serial dilution method employing 96-well flat-bottomed microtitration plates inoculated with *C. acnes* strains at a final concentration of 1.0 × 10^5^ colony-forming units (CFU)/mL. After incubation, the microtitration plates were read visually at an optical density of 600 nm (OD_600_), and the concentration of the chitosan–phytochemical conjugate that exhibited no turbidity was recorded as the MIC. MBCs were the lowest concentration to kill an initial bacterial inoculum by ≥99.9%; they can be determined from broth dilution MIC tests by sub-culturing to agar plates [23].

### 2.5. C. acnes-Induced Inflammation in Mice

Eight-week-old male ICR mice were purchased from Charles River Laboratories (Wilmington, MA, USA) and maintained in our specific pathogen-free animal facility. All protocols used in the animal studies were approved by the Animal Care and Use Committee of the College of Medicine, Inje University, Korea, and all experiments were performed according to appropriate ethical guidelines (IACUC Approval No. 2015-036). We determined the in vivo anti-acne vulgaris effects of SMYEE using *C. acnes*-injected mice. Three different regions of the dorsal skin of each of six mice were labeled CON (control, phosphate-buffered saline (PBS) + Vaseline (VAS)), CA (*C. acnes* (CA) + VAS), and SMYEE (CA + VAS + SMYEE). *C. acnes* (1 OD_600_ unit/10 μL in PBS) was intradermally injected into two spots on each mouse, one of which received Vaseline (50 mg) applications for 6 days (CA) and the other SMYEE (50 μg) mixed with 50 mg Vaseline (Sigma) for 6 days (SMYEE).

### 2.6. Histopathology

Seven days after *C. acnes* inoculation, the skin nodules were excised, initially stored in 4% (*v*/*v*) paraformaldehyde, embedded in paraffin wax, cut into 4-μm thick sections, stained with hematoxylin and eosin, and examined using a digital slide scanner (NanoZoomer 2.0-RS; Hamamatsu, Shizuoka, Japan).

### 2.7. Immunohistochemistry

Four-micrometer thick paraffin-embedded tissue sections were incubated overnight with an anti-NIMP-R14 antibody, and NIMP-R14 levels were evaluated using the NanoZoomer 2.0-RS instrument.

### 2.8. Cell Culture

Human keratinocyte HaCaT cells were purchased from the Korean Cell Line Bank (Seoul, Korea) and grown in the Dulbecco’s modified Eagle medium (Gibco, Grand Island, NY, USA) supplemented with 10% (*v*/*v*) fetal bovine serum (FBS) and 100 units/mL penicillin-streptomycin antibiotics (Gibco, Gaithersburg, MD, USA) at 37 °C in a humidified incubator containing a 5% (*v*/*v*) CO_2_ atmosphere. HL-60 cells were maintained in RPMI-1640 medium supplemented with 10% (*v*/*v*) FBS and induced to differentiate into dHL-60 cells by exposure to 1.75% (*v*/*v*) dimethyl sulfoxide for 3–4 days to trigger expression of the neutrophilic phenotype.

### 2.9. Cell Viability

Cell viability was evaluated using the Cell Counting Kit-8 (CCK-8) assay method (Dojindo Molecular Technologies Inc., Gaithersburg, MD, USA). Briefly, 96-well microplates containing HaCaT cells were cultivated in Dulbecco’s Modified Eagle’s Medium (DMEM). The cells were incubated with various concentrations of SMYEE for 24 h at 37 °C. Next, the cells were incubated with CCK-8 for 1 h. Absorbance at 450 nm was recorded using a microplate reader (SpectraMax M2e; Molecular Devices, Sunnyvale, CA, USA). All assays were performed in triplicate.

### 2.10. Enzyme-Linked Immunosorbent Assay

IL-8 levels in cell culture media were measured using an ELISA kit (R&D Systems, Minneapolis, MN, USA). Absorbance at 450 nm was recorded using a SpectraMax M2e instrument.

### 2.11. Western Blotting

Cells were lysed in a lysis buffer (1% (*v*/*v*) Triton X-100, 1% (*w*/*v*) sodium deoxycholate, and 0.1% [*w*/*v*] NaN_3_) with a protease inhibitor cocktail (Roche Diagnostics, Mannheim, Germany). Each skin sample was ground in liquid nitrogen, and 0.1 mg amounts of powder were lysed; 20-μg protein samples were subjected to Western blotting. Nuclear proteins were prepared using a nuclear extraction reagent (Pierce Biotechnology, Inc., Rockford, IL, USA) according to the manufacturer’s instructions. Equal quantities of protein were separated on 10% (*w*/*v*) sodium dodecyl sulfate-polyacrylamide mini-gels and transferred to nitrocellulose membranes. After overnight incubation with specific antibodies, membranes were incubated with a HRP-conjugated secondary antibody at room temperature for 1 h. Band staining intensity was quantified using an enhanced chemiluminescence detection system (Pierce) and Multi Gauge software (ver. 2.2; Fuji Film, Tokyo, Japan).

### 2.12. Cell Migration

Migration of dHL-60 cells was evaluated using a 24-well plate Transwell system (Becton-Dickinson, Franklin Lakes, NJ, USA) at a cell density of 5 × 10^5^ cells/mL. dHL-60 cells were added to upper chambers separated from lower chambers by 3-μm-pore-diameter filters. *C. acnes*-stimulated HaCa T cells were added to the lower chambers. dHL-60 cells were allowed to migrate toward the lower chambers for 48 h at 37 °C in a 5% (*v*/*v*) CO_2_ atmosphere. Migrated cells in the lower chambers were collected via centrifugation at 400 g for 10 min and counted using a hemocytometer. Each experiment was performed in triplicate and repeated at least three times.

### 2.13. Electrophoretic Mobility Shift Assay

Nuclear extracts were prepared using the NE-PER™ nuclear extraction reagent (Pierce). The probe for the gel retardation assay was an oligonucleotide containing the AP-1 binding site (5′-CGC TTG ATG ACT CAG CCG GAA-3′) purchased from Santa Cruz Biotechnology. The 3′ end of the probe was labeled with biotin (Pierce). Each competitive reaction proceeded via the addition of a 100-fold excess of unlabeled AP-1 to the reaction mixture, which was then subjected to electrophoresis on a 5% (*w*/*v*) polyacrylamide gel using 0.5× Tris-borate buffer and transferred to nylon membranes. Biotin-labeled DNA was detected using the LightShift chemiluminescent electrophoretic mobility shift assay (EMSA) kit (Pierce).

### 2.14. Statistical Analysis

Data are presented as mean ± standard error of mean (SEM). All statistical analyses were performed using the GraphPad Prism software (ver. 5.0; GraphPad Software Inc., La Jolla, CA, USA). Between-group comparisons were performed using the Dunnett’s multiple range test. *p* < 0.05 was considered statistically significant.

## 3. Results

### 3.1. Antibacterial Effects of SMYEE

The antibacterial activity of SMYEE against *C. acnes* strains was evaluated by the determination of minimum inhibitory concentrations (MICs) and minimum bactericidal concentrations (MBCs) (Table 1). The MICs ranged from 128 µg/mL to 512 µg/mL and the MBCs ranged from 512 µg/mL to 4096 µg/mL. Thus, SMYEE showed inhibitory activity towards *C. acnes*; to the best of our knowledge, this is the first report to demonstrate such activity.

### 3.2. Effects of SMYEE on C. acnes-Induced Clinical Changes

**Figure 1:**
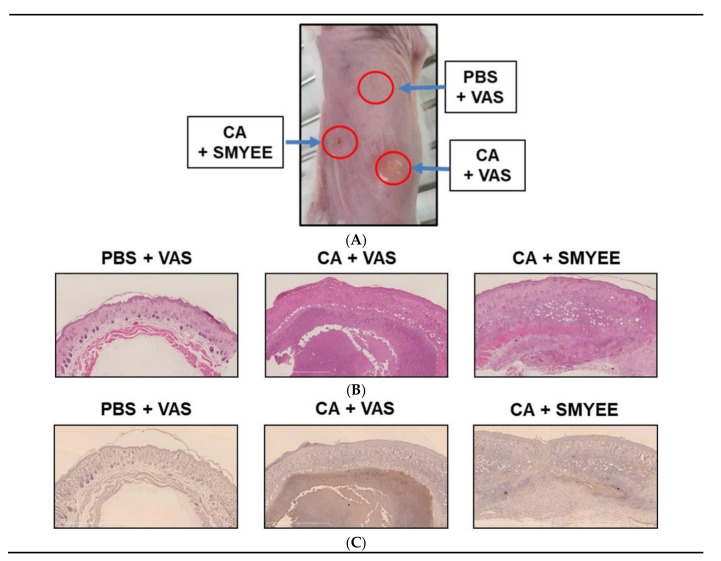
The effects of *Sargassum miyabei* Yendo ethanolic extract (SMYEE) on inflammation in a model of *Cutibacterium acnes* (*C. acnes*)-induced inflammatory skin disease. The dorsal skin of mice was intradermally injected with 10 μL (optical density at 600 nm [OD_600_] = 1.0 mL) of *C. acnes*. SMYEE (50 μg mixed with 50 mg Vaseline) was then applied to the skin. (**A**) The inflammatory lesions; (**B**) hematoxylin and eosin staining; (**C**) Neutrophil infiltration of dorsal skin was examined by immunohistochemical staining for NIMP-R14 (a neutrophil marker). CA: *C. acnes*, VAS: Vaseline, PBS: phosphate-buffered saline

We explored the therapeutic effects of SMYEE in the context of *C. acnes*-induced clinical changes. *C. acnes* was intradermally injected into the dorsal skin of ICR mice, and 50 μg SMYEE mixed with 50 mg Vaseline was applied to the lesions twice daily for 6 days. As shown in Figure 1A, significant nodule development, skin swelling, redness, and erythema were observed 7 days after *C. acnes* injection. Histologically, *C. acnes* induced epidermal hyperplasia and thickening, as well as marked accumulation of inflammatory cells (Figure 1B). Thus, SMYEE clearly reduced these inflammatory events.

### 3.3. SMYEE Suppressed Neutrophil Accumulation in Inflammatory Lesions

*C. acnes* significantly elevated neutrophil levels in acne lesions. We explored whether SMYEE attenuated neutrophil infiltration into such lesions. Immunohistochemical staining for NIMP-R14, a neutrophil marker, revealed neutrophil infiltration into the inflammatory lesions. As shown in Figure 1C, *C. acnes* lesions (CA + Vaseline (VAS)) exhibited significantly more NIMP-R14-positive cells compared to those in the control (CON regions: phosphate-buffered saline (PBS) + VAS). SMYEE (CA + SMYEE) significantly reduced the number of NIMP-R14-positive cells in the *C. acnes* lesions. SMYEE thus suppressed the inflammatory response of acne vulgaris in our animal model.

### 3.4. Effect of SMYEE on HaCaT Cell Viability

**Figure 2:**
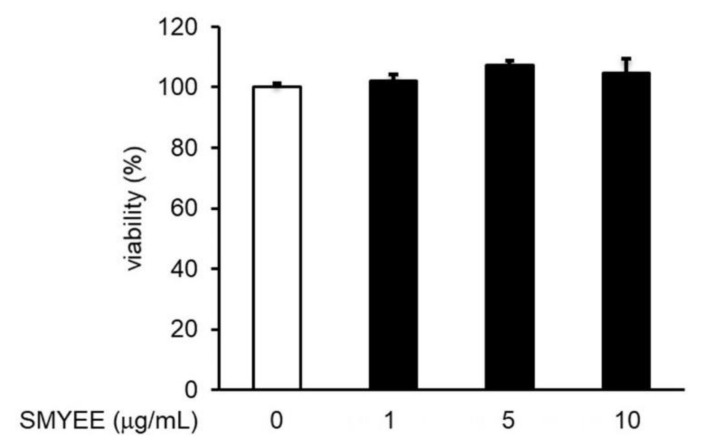
Effects of *Sargassum miyabei* Yendo ethanolic extract (SMYEE) on HaCaT cell viability. Cells were treated with various concentrations (1–10 µg/mL) of SMYEE for 24 h. Cell viability was assessed using the Cell Counting Kit-8 (CCK-8) assay. The results are expressed as the percentages of surviving cells relative to those of untreated cells. The data are means ± standard error of the mean (SEM) and are representative of the results obtained in three independent experiments.

The viability of HaCaT cells treated with SMYEE was evaluated using the Cell Counting Kit-8 (CCK-8) assay. SMYEE was non-toxic to HaCaT cells at levels under 10 μg/mL (Figure 2). We thus tested SMYEE over the range of 1–10 µg/mL.

### 3.5. Effects of SMYEE on IL-8 Levels in C. acnes-Stimulated HaCaT Cells

**Figure 3:**
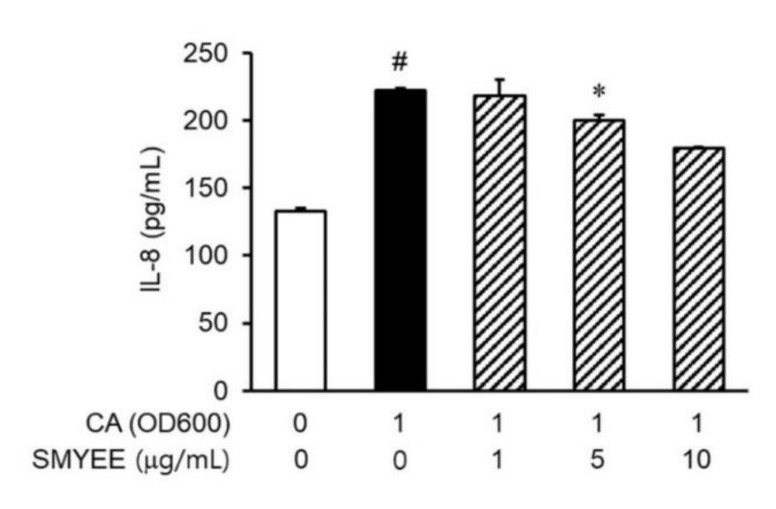
Effects of *Sargassum miyabei* Yendo ethanolic extract (SMYEE) on interleukin (IL)-8 production in *Cutibacterium acnes* (*C. acnes*)-induced HaCaT cells seeded at 2 × 10^5^ cells/mL and incubated with various concentrations (1 µg/mL, 5 µg/mL, and 10 µg/mL) of SMYEE for 30 min, followed by *C. acnes* stimulation (1 optical density unit at 600 nm [OD_600_]). After stimulation with *C. acnes* for 24 h, IL-8 production by HaCaT cells was determined via enzyme-linked immunosorbent assay (ELISA) using the supernatant. The data are means ± standard error of the mean (SEM) and are representative of the results obtained in three independent experiments. ^#^ *p* < 0.05 vs. the no-treatment group; * *p* < 0.05 vs. the *C. acnes* group. CA: *C. acnes*.

IL-8 levels in HaCaT cells increased considerably after stimulation by *C. acnes* (Figure 3). The inhibitory effects of SMYEE on *C. acnes*-induced IL-8 production in HaCaT cells were determined by an enzyme-linked immunosorbent assay (ELISA). SMYEE (1 µg/mL, 5 µg/mL, or 10 μg/mL for 30 min prior to *C. acnes* stimulation for 24 h) suppressed *C. acnes*-induced IL-8 production.

### 3.6. Effects of SMYEE on dHL-60 Cell Migration

**Figure 4:**
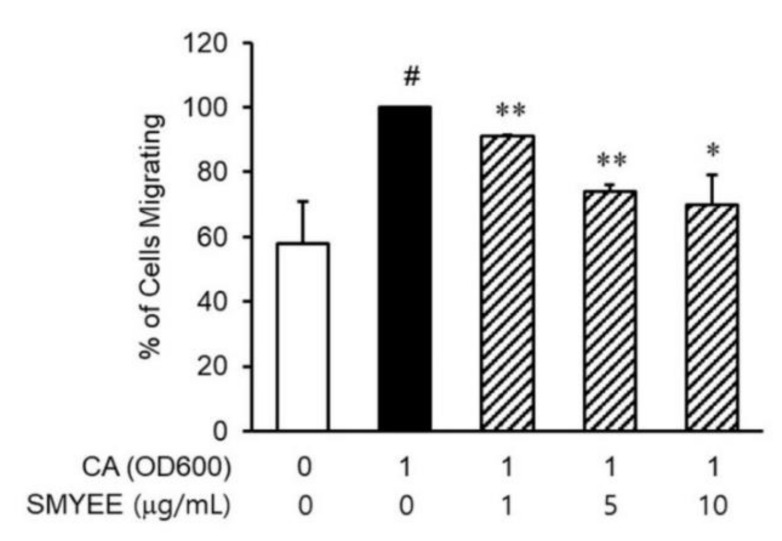
Effects of *Sargassum miyabei* Yendo ethanolic extract (SMYEE) on in vitro migration of human neutrophil-like HL-60 (dHL-60) cells. The cells were added to the upper chambers of Transwell cluster plates separated from the lower chambers by 3-μm-pore-diameter filters. *Cutibacterium acnes* (*C. acnes*)-induced HaCaT cells were added to the lower chambers. A hemocytometer was used to count the number of dHL-60 cells that migrated to the lower chambers. The data are means ± standard error of the mean (SEM) and are representative of the results obtained in three independent experiments. # *p* < 0.05 vs. the no-treatment group; * *p* < 0.05 and ** *p* < 0.005 vs. the *C. acnes* group. CA: *C. acnes*.

Neutrophils are recruited to inflammatory lesions by their attraction to chemokines. To explore whether SMYEE affected the migration of dHL-60 cells, migration assays were performed using Transwell cluster plates (Figure 4). The number of migrated *C. acnes*-induced dHL-60 cells was greater than that of vehicle-treated dHL-60 cells and was significantly lower in the SMYEE group compared to the SMYEE-free *C. acnes*-induced control group.

### 3.7. Effects of SMYEE on Extracellular Signal-Regulated Kinase (ERK) Signaling Pathways in C. acnes-Stimulated HaCaT Cells

**Figure 5:**
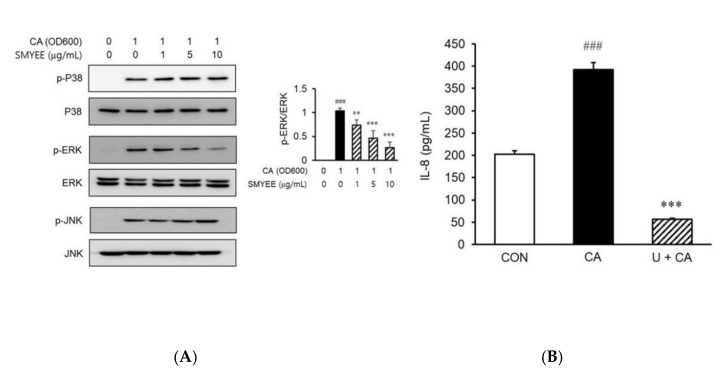
Effects of *Sargassum miyabei* Yendo ethanolic extract (SMYEE) on mitogen-activated protein kinase (MAPK) phosphorylation in *Cutibacterium acnes* (*C. acnes*)-induced HaCaT cells treated with the indicated concentrations of SMYEE for 30 min, followed by stimulation with *C. acnes* (1 optical density at 600 nm [OD_600_] unit) for 15 min. (**A**) Cytosolic proteins were subjected to Western blotting using antibodies specific for the phosphorylated forms of p38 MAPK, extracellular signal-regulated kinase (ERK), and c-Jun N-terminal kinase (JNK). ^###^ *p* < 0.001 vs. the no-treatment group; ** *p* < 0.005 and *** *p* < 0.001 vs. the *C. acnes* group. (**B**) After treatment with *C. acnes* for 24 h in the presence or absence of U0126 (10 μM) for 30 min, cell supernatants were isolated, and interleukin (IL)-8 levels were assayed by enzyme-linked immunosorbent assay (ELISA). The data are means ± standard error of the mean (SEM) and are representative of the results obtained in three independent experiments. ### *p* < 0.001 vs. the no-treatment group; *** *p* < 0.001 vs. the *C. acnes* group. CON: no treatment, CA: *C. acnes*, U: U0128.

To clarify the mechanisms underlying the effects of SMYEE on IL-8 production, we evaluated the activation status of mitogen-activated protein kinases (MAPKs) by Western blotting. Stimulation by *C. acnes* of HaCaT cells increased the phosphorylation of ERK, c-Jun N-terminal kinase (JNK), and p38 MAPK. However, pretreatment for 30 min with SMYEE (1 µg/mL, 5 µg/mL, or 10 µg/mL) attenuated ERK phosphorylation induced by a 15-min incubation with 1 OD_600_ unit of *C. acnes* (Figure 5A), but did not affect the phosphorylation of p38 MAPK or JNK. To confirm that the ERK signaling pathway was involved in IL-8 production, HaCaT cells were treated with *C. acnes* with or without the ERK inhibitor, U0126. The increase in IL-8 production was significantly attenuated by the inhibitor (Figure 5B). Thus, SMYEE suppressed IL-8 production by inhibiting the ERK pathway.

### 3.8. Effects of SMYEE on Activator Protein-1 (AP-1) Activation in C. acnes-Stimulated HaCaT Cells

**Figure 6:**
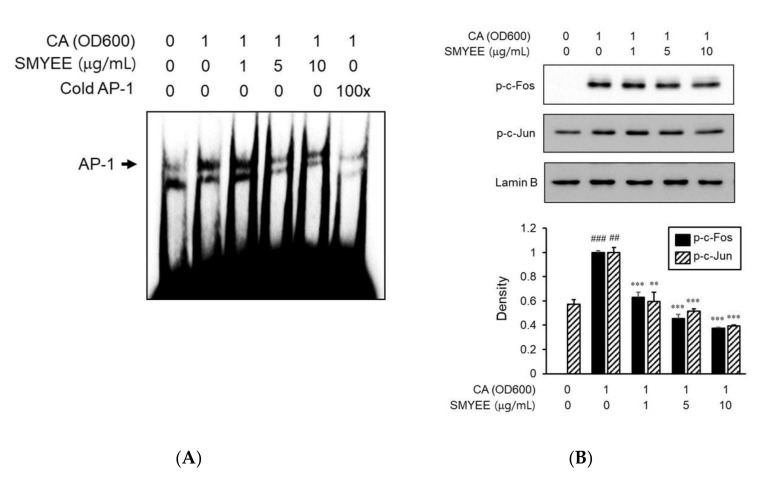
Effects of *Sargassum miyabei* Yendo ethanolic extract (SMYEE) on activator protein-1 (AP-1) activity in *Cutibacterium acnes* (*C. acnes*)-induced HaCaT cells. Cells were pretreated with SMYEE (1–10 µg/mL) for 30 min prior to stimulation with *C. acnes* (1 optical density at 600 nm [OD_600_] unit) for 1 h. Nuclear extracts were analyzed in terms of AP-1 activity. (**A**) Electrophoretic mobility shift assay (EMSA) showing that SMYEE attenuated the DNA-binding activity of AP-1 in *C. acnes*-induced HaCaT cells. (**B**) Nuclear proteins were isolated, and c-Fos and c-Jun nuclear translocation levels were analyzed by Western blotting. The data are means ± standard error of the mean (SEM) and are representative of the results obtained in three independent experiments. ^##^ *p* < 0.005 and ^###^ *p* < 0.001 vs. the no-treatment group; ** *p* < 0.005 and *** *p* < 0.001 vs. the *C. acnes* group. CA: *C. acnes*.

To explore the mechanism of nuclear transcription by which SMYEE affected IL-8 production, we assessed the effects of SMYEE on AP-1 activation. We explored the effect of SMYEE on the DNA-binding activity of AP-1 using an electrophoretic mobility shift assay (EMSA) (Figure 6A). *C. acnes* induction significantly increased the DNA-binding activity of AP-1; SMYEE (5 and 10 g/mL) pretreatment prevented this increase. We next examined the nuclear localization status of phosphorylated c-Fos and c-Jun proteins in SMYEE-pretreated HaCaT cells exposed to *C. acnes* stress. SMYEE inhibited the *C. acnes*-induced nuclear translocation of phosphorylated c-Fos and c-Jun (Figure 6B). Thus, *C. acnes*-induced activation of the AP-1 pathway was inhibited by SMYEE.

## 4. Discussion

Acne vulgaris is the most common inflammatory skin disease worldwide, characterized by the formation of inflammatory nodules, pustules, and papules. The condition affects ~85% of adolescents worldwide and can persist into adulthood, adversely affecting the quality of life by inducing depression, anxiety, self-isolation, and (in extreme cases) suicidal thoughts, as well as physical scarring [17]. Effective acne treatments are urgently needed. In a previous study, *Sargassum miyabei Yendo* exhibited anti-inflammatory activity [24]. Thus, we evaluated whether SMYEE exerted antibacterial effects in an animal model of acne, and anti-inflammatory effects in *C. acnes*-induced HaCaT cells, in the absence of any toxicity.

Seaweeds are important food and medicinal resources. A previous study found that a methanolic extract of the edible seaweed, *Eisenia bicyclis*, exhibited antibacterial activity against *C. acnes* at 512–1024 µg/mL [25]. We first explored whether SMYEE was active against *C. acnes* by determining the MICs. SMYEE inhibited *C. acnes* growth with MICs of 128–512 μg/mL. Thus, we concluded that SMYEE might be a safe and effective acne treatment. We evaluated the effects of SMYEE on the *C. acnes*-induced inflammatory response in vivo using a well-known mouse acne model; the lesions are similar to those of humans [26]. *C. acnes* injection into mouse dorsal skin induces nodule development, swelling, redness, erythema, epidermal hyperplasia and thickening, as well as the accumulation of inflammatory cells (especially neutrophils). SMYEE significantly attenuated all of these responses and, hence, may be a useful acne treatment.

It is well known that *C. acnes* induces the release of proinflammatory cytokines and chemokines, including IL-1β, IL-6, interferon (IFN)-γ, TNF-α, and IL-8 at the onset of infection [27]. Of these, IL-8 is a chemoattractant that promotes neutrophil migration into inflammatory lesions, such as skin pilosebaceous units. After bacterial infection, neutrophils rapidly recognize and clear bacteria. Thus, we focused on whether neutrophils accumulated in acne lesions, and whether IL-8 was produced in such lesions. In vivo, we used immunohistochemistry to show that neutrophils accumulated in the lesions. Therefore, control of IL-8 production may reliably and effectively treat acne. In vitro, *C. acnes* induced IL-8 secretion by HaCaT cells and induced migration of dHL-60 neutrophil-like cells to regions containing HaCaT cells. However, SMYEE attenuated both IL-8 secretion by *C. acnes*-induced HaCaT cells and the migration of dHL-60 cells, suggesting that SMYEE inhibition of chemotaxis (which attenuated *C. acnes*-induced neutrophil migration to infected lesions) was mediated by inhibition of IL-8 secretion.

It is well known that the MAPK pathways (the JNK, ERK, and p38 pathways) are associated with the *C. acnes*-induced inflammatory responses of HaCaT cells [28,29]. MAPKs play important roles in the production of proinflammatory cytokines. We found that *C. acnes* increased MAPK phosphorylation in HaCaT cells. SMYEE decreased *C. acnes*-stimulated ERK phosphorylation, but did not affect JNK or p38 MAPK phosphorylation status. As expected, IL-8 production was suppressed by an ERK inhibitor (U0126) after its *C. acnes*-mediated induction in HaCaT cells. Thus, SMYEE inhibited the *C. acnes*-stimulated inflammatory responses that are mediated by the ERK signaling pathway. We next examined downstream ERK-mediated activities. In previous studies, IL-8 production increased upon activation of various transcription factors, including nuclear factor-kappa B (NF-κB) and AP-1, which are involved in many pathophysiological responses including the inflammation response seen in various skin diseases [30,31,32]. We used an EMSA to explore NF-κB and AP-1 activation status. NF-κB and AP-1 production was activated by *C. acnes*. However, AP-1 transactivation induced by *C. acnes* was attenuated by SMYEE, but NF-κB activation was not (data not shown). As AP-1 regulated the inflammatory response of interest, we observed nuclear activation of c-Jun and c-Fos (the latter is an AP-1 subunit), as expected. SMYEE attenuated the *C. acnes*-stimulated nuclear phosphorylation of c-Fos and c-Jun. Together with our mechanistic data, the results indicate that SMYEE inhibited *C. acnes*-induced AP-1 activation by suppressing ERK signaling in vitro.

In conclusion, SMYEE effectively protected against *C. acnes*-induced responses in an in vivo acne-like model. SMYEE pretreatment suppressed the production of inflammatory cytokines by *C. acnes*-induced keratinocytes. SMYEE blocked AP-1 activation by modulating the ERK signaling pathway. Thus, SMYEE may be useful for treating inflammatory skin disorders in patients with *C. acnes* infections. SMYEE is expected to contribute to the development of alternative phytotherapeutic agents without side effects on the human body caused by antibiotics or steroids. Therefore, the relevant bioactive compounds of SMYEE, natural derived therapeutic substances with less side effects, should be isolated and evaluated. We will make a fraction (n-hexane, chloroform, ethyl acetate, n-butanol) according to the polarity of the solvent and confirm the activity for this fraction. In addition, it is planned to separate the active compound by chromatography on an effective fraction.

## Figures and Tables

**Table 1:** Minimum inhibitory concentration (MIC) and minimum bactericidal concentration (MBC) of *Sargassum miyabei Yendo* ethanol extract (SMYEE) against *Cutibacterium acnes* (*C. acnes*) strains.

Strains	MIC (µg/mL)	MBC (µg/mL)
*C. acnes* KCTC 3314	256	512
*C. acnes* isolates 2874	256	2048
*C. acnes* isolates 2875	256	1024
*C. acnes* isolates 2876	128	1024
*C. acnes* isolates 2878	512	4096
